# Administration of Blood Salts

**Published:** 1907-07

**Authors:** 


### Administration of Blood Salts.

Privy Councillor Senator says in the *Russische med. Rundschau,* Jan., 1907, that antisclerosin is the simplest and most convenient method of administering the blood salts, the use of which is based on the work of Dennstedt-Rumph and Ludwig Weil. The antisclerosin tablets contain chiefly sodium chloride, with sodium sulphate, sodium phosphate, sodium carbonate, magnesium phosphate and calcium glycerophosphate,—all in about the proportion in which these salts are normally present in the blood. The initial dose is two tablets daily and is gradually increased to six tablets daily. If the dosage is slowly raised, antisclerosin is well borne. Though the casual relation of salt impoverishment of the blood to arteriosclerosis is not yet wholly explained, salt impoverishment is certainly an abnormal condition. The careful use of antisclerosin can only be of benefit.

